# Unraveling Parkinson's disease: The mystery of mitochondria and the role of aging

**DOI:** 10.1016/j.gendis.2025.101719

**Published:** 2025-06-10

**Authors:** Tingting Liu, Jingwen Li, Haojie Wu, Junbo Qiao, Jianshe Wei

**Affiliations:** a Institute for Brain Sciences Research, School of Life Sciences, Henan University, Kaifeng, Henan 475004, China; b Institute for Sports and Brain Health, School of Physical Education, Henan University, Kaifeng, Henan 475004, China; c Department of Vascular Tumors, Third Affiliated Hospital of Zhengzhou University, Zhengzhou, Henan 450052, China

**Keywords:** Aging, Astrocytes, Microglia, Mitochondrial dysfunction, Parkinson's disease

## Abstract

Parkinson's disease (PD) is a complex neurodegenerative disorder that poses significant burden on patients and families. Its exact cause is unknown, resulting in limited effective treatments. Mitochondrial dysfunction, linked to genetics, aging, oxidative stress, and environmental factors, is central to PD. Healthy elderly individuals have a compensatory mitochondrial DNA (mtDNA) mechanism in brain cells, but this mechanism is impaired in PD patients, leading to mtDNA reduction, respiratory chain dysfunction, decreased adenosine triphosphate (ATP) synthesis, and inadequate neuron energy. Aging increases oxidative stress, impairing mitochondrial function. Mitochondrial dysfunction in the dopaminergic neurons of the substantia nigra causes neuronal loss and disease progression. Aging microglia also play a crucial role, with a reduced capacity to clear neurotoxic substances, especially in the substantia nigra. A decrease in triggering receptor expressed on myeloid cells 2 (TREM2) gene expression shifts microglia to a pro-inflammatory phenotype, exacerbating neuroinflammatory responses and protein deposition. Down-regulation of the C-X3-C motif chemokine ligand 1 (CX3CL1)/C-X3-C chemokine receptor 1 (CX3CR1) signaling pathway increases the expression of pro-inflammatory cytokines, accelerating neuronal loss and disease progression. Recent research has identified a new astrocyte aging regulatory mechanism involving the cyclic GMP‒AMP synthase (cGAS)/stimulator of interferon genes (STING) signaling pathway, promoting astrocyte aging and exacerbating dopamine neuronal loss and motor dysfunction. Understanding PD pathogenesis, especially mitochondrial dysfunction, aging, and glial cell changes, is crucial for developing effective treatments.

## Introduction

**Figure 1:**
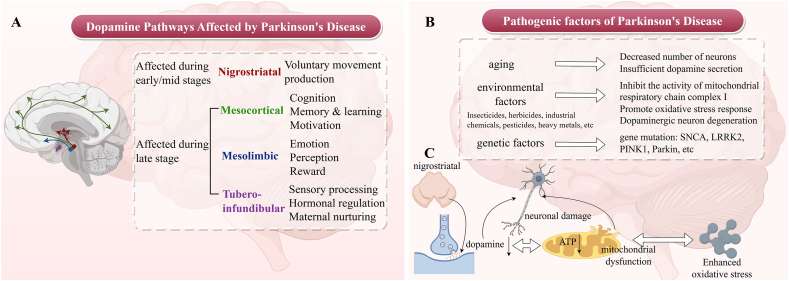
Affected brain regions and pathogenic factors of PD. **(A)** In early/mid-stage PD, the nigrostriatal pathway (indicated by red arrow) is primarily affected, leading to impaired voluntary movement control due to dopamine depletion in the striatum. During late-stage PD, three additional pathways are compromised: the mesocortical pathway (green arrow), associated with cognitive functions such as memory and motivation; the mesolimbic pathway (blue arrow), involved in emotional processing and reward perception; and the tubero-infundibular pathway (purple arrow), which regulates hormonal balance and sensory integration. **(B)** The pathogenesis of PD involves three major factors: (1) aging, which accelerates mitochondrial dysfunction (2) environmental toxins (e.g., pesticides, heavy metals) that inhibit mitochondrial complex I and induce oxidative stress; and (3) genetic mutations (e.g., *SNCA*, *LRRK2*, *PINK1*, and *Parkin*) that disrupt mitochondrial quality control and autophagy. (C) The cascade of neuronal damage begins with dopaminergic neuron degeneration in the substantia nigra, causing insufficient dopamine secretion. Mitochondrial dysfunction exacerbates this process by reducing ATP production and amplifying oxidative stress, creating a vicious cycle that further damages neurons and drives disease progression.

Parkinson's disease (PD) is a common neurodegenerative disorder primarily affecting the motor nervous system,^1^ with a pathogenesis that progresses in distinct stages. In the early stage, degeneration of dopaminergic neurons in the nigrostriatal pathway reduces dopamine levels in the striatum, disrupting the excitatory‒inhibitory balance and leading to classic motor symptoms such as resting tremor and muscle rigidity. As the disease progresses to the early-to-mid stages, involvement of the mesocortical pathway leads to prefrontal cortex dysfunction due to dopamine depletion, causing cognitive decline; simultaneously, impairment of the mesolimbic pathway induces emotional dysregulation and blunted reward perception (Fig. 1A). Although the tubero-infundibular pathway is affected in late stages, its associated endocrine symptoms are less clinically prominent than motor and cognitive manifestations.^2^, ^3^, ^4^

The etiology of PD involves multiple factors, including aging, environmental exposure, and genetic susceptibility,^5^, ^6^, ^7^ with mitochondrial dysfunction serving as a central pathogenic hub linking these elements. Age-related mitochondrial decline induces dopaminergic neuron damage through oxidative stress and mitochondrial DNA (mtDNA) mutations by impairing oxidative phosphorylation, reducing adenosine triphosphate (ATP) production, and increasing reactive oxygen species (ROS) generation (Fig. 1B). Environmental toxins such as pesticides (e.g., rotenone and paraquat) and heavy metals specifically target mitochondrial complex I, disrupting the integrity of the electron transport chain. This dysfunction not only exacerbates energy deficit but also triggers the opening of mitochondrial permeability transition pores, initiating apoptotic cascades.^8^ Mutations in PD-associated genes, such as α-synuclein (*SNCA*), leucine-rich repeat kinase 2 *(LRRK2*), PTEN-induced putative kinase 1 (*PINK1*), and *Parkin*, directly impact mitochondrial quality control: PINK1 and Parkin mutations disrupt mitophagy, the selective degradation of damaged mitochondria; *SNCA* oligomers accumulate in mitochondria, impairing membrane integrity; and LRRK2 dysregulation affects mitochondrial dynamics, leading to fragmented and dysfunctional organelles.^9^, ^10^, ^11^, ^12^

Mitochondrial dysfunction and oxidative stress form a pathogenic cycle in PD: Damaged mitochondria fail to buffer calcium homeostasis and produce insufficient ATP, while excessive ROS generation damages cellular components, including mitochondrial membranes and DNA. This vicious cycle selectively affects dopaminergic neurons due to their high energy demand and intrinsic vulnerability to oxidative injury^13^^,^^14^ (Fig. 1C). Given the central role of mitochondria in integrating aging, environmental, and genetic insults, this review focuses on how mitochondrial bioenergetic failure, dynamic abnormalities, and mitophagic dysfunction interact with aging processes to drive PD progression, with a particular emphasis on glial cell-mediated mitochondrial dysfunction and potential therapeutic strategies targeting mitochondrial pathways.

A systematic literature search was conducted via PubMed, Web of Science, and Google Scholar, focusing on studies published between 2000 and 2024. Keywords included PD, mitochondrial dysfunction, aging, microglia, and related terms. After removing duplicates and filtering titles/abstracts, articles that meet the inclusion criteria (relevance to PD mechanisms) were identified. The full-text review excluded studies lacking experimental rigor, resulting in 150 cited references (Fig. S1).

## Factors leading to mitochondrial dysfunction

Mitochondrial dysfunction plays a crucial role in the pathogenesis of PD, with contributing factors including genetic susceptibility, aging, and oxidative stress. PD is now recognized as a neurodegenerative disease caused by multiple factors, and its pathogenesis is closely related to mitochondrial dysfunction. From a genetic perspective, PD demonstrates a familial tendency in some families. Mutations in genes such as *SNCA*, *LRRK2*, *PINK1*, and *Parkin* significantly increase the risk of PD among carriers, and these genetic mutations are often closely linked to damage to mitochondrial function and subsequent degradation of damaged mitochondria.^15^ For example, the proteins encoded by the *PINK1* and *Parkin* genes play key roles in normal mitochondrial quality control mechanisms, marking damaged mitochondria and initiating autophagic degradation to maintain the health of the mitochondrial population within the cell. However, when these genes mutate, damaged mitochondria cannot be normally cleared, leading to their accumulation and release of neurotoxic substances that damage neurons and ultimately promote the occurrence and progression of PD.^16^^,^^17^

Aging is also a factor that cannot be ignored. With age, the antioxidant defense system of the body gradually declines, and the production of oxidants such as free radicals increases, elevating the level of oxidative stress. Excessive free radicals attack mitochondrial membranes, mtDNA, and other cellular macromolecules.^18^ In PD, dopaminergic neurons in the substantia nigra are sensitive to oxidative stress. When mitochondria are attacked by free radicals, their function fails, leading to changes in the membrane potential and decreased activity of respiratory chain complexes, and further resulting in reduced ATP synthesis and insufficient energy supply to neurons. At the same time, more oxidative products are produced, forming a vicious cycle that leads to the death of a large number of dopaminergic neurons and the gradual emergence and aggravation of PD symptoms. Moreover, during aging, mitochondrial function itself declines, with phenomena such as reduced activity of internal metabolic enzymes, altered membrane permeability, and accumulation of DNA mutations becoming more common. These changes impair the originally efficient energy production and ion balance maintenance functions of mitochondria.^19^^,^^20^ Especially in the dopaminergic neurons of the substantia nigra, once mitochondrial function fails, the electrical activity of neurons, the synthesis and release of the neurotransmitter dopamine, and other processes will be severely impaired, ultimately promoting disease progression.

In terms of oxidative stress, an imbalance between oxidants and antioxidants in the body leads to cellular damage. Enhanced oxidative stress responses in PD patients can damage and even kill dopaminergic neurons in the substantia nigra, which is related to excessive free radical production or reduced antioxidant levels. Furthermore, mitochondrial dysfunction and oxidative stress interact with each other. Dysfunctional mitochondria can further increase oxidative stress, and conversely, increased oxidative stress continuously exacerbates mitochondrial damage. Together, they continuously impair neurons and accelerate the deterioration of PD.^21^^,^^22^ Mutations in the PD-related gene *PARK7* alter the structure and function of the encoded protein DJ-1, leading to an autosomal recessive genetic disorder that increases the risk of PD. DJ-1 was first discovered as an oncogene in 1997^23^ and was associated with early-onset PD in 2003.^24^ DJ-1 mutations account for about 1% of all recessive genetic early-onset PD cases, and the function of this protein has been extensively studied. In healthy subjects, DJ-1 serves as an antioxidant and oxidative stress sensor in various neuroprotective mechanisms. It also participates in mitochondrial homeostasis, apoptosis regulation, chaperone-mediated autophagy (CMA), and dopamine homeostasis by regulating various signaling pathways, transcription factors, and chaperone functions. While DJ-1 protects neurons from reactive oxygen species, neurotoxins, and mutant α-synuclein, protein mutations may lead to inefficient neuroprotection and the progression of PD.^25^

In addition to genetic and aging factors, environmental factors also play a significant role in mitochondrial dysfunction and the pathogenesis of PD.^26^^,^^27^ Long-term exposure to various environmental substances can have detrimental effects on mitochondrial function, ultimately increasing the risk of PD. Pesticides, herbicides, and rotenone are threatening substances. Rotenone inhibits mitochondrial complex I, disrupting electron flow and increasing ROS production. Excessive ROS cause oxidative damage and α-synuclein aggregation.^28^ Chronic rotenone exposure in models replicates PD pathology, including nigrostriatal degeneration, which leads to dopamine-related symptoms. Heavy metals like lead and mercury affect mitochondrial function. Lead disrupts mitochondrial Ca^2+^ homeostasis, causing mitochondrial dysfunction. Mercury binds to superoxide dismutase (SOD), inhibiting its antioxidant activity and exacerbating oxidative stress, contributing to PD.^29^ 1-Methyl-4-phenyl-1,2,3,6-tetrahydropyridine (MPTP) is another toxin. Metabolized to MPP^+^, it accumulates in dopaminergic neurons via dopamine transporters. MPP^+^ inhibits mitochondrial complex I, increasing ROS and inducing apoptosis.^30^ Dopaminergic neuron death is a PD hallmark, and MPTP-induced damage helps understand the disease mechanisms.

Overall, these environmental factors can independently or interactively cause mitochondrial dysfunction, which is a central event in the pathogenesis of PD. Understanding the complex relationships among environmental exposure, mitochondrial function, and PD development is crucial for developing preventive strategies and more effective treatments for this debilitating neurodegenerative disorder.

## Mitochondrial dysfunction in PD

**Figure 2:**
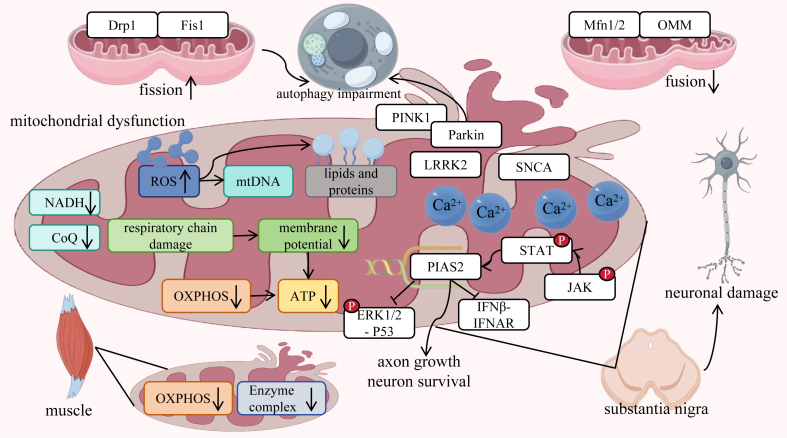
Molecular mechanisms of mitochondrial dysfunction in PD. (1) imbalances in mitochondrial dynamics, where excessive fission (mediated by Fis1/Drp1) and impaired fusion disrupt organelle integrity; (2) impaired autophagy, evidenced by defective PINK1/Parkin-mediated mitophagy, leading to the accumulation of damaged mitochondria; (3) oxidative stress, manifested through respiratory chain damage (NADH/CoQ dysfunction), mtDNA mutations, and lipid/protein oxidation; (4) energy metabolism collapse, with reduced ATP production due to OXPHOS defects in both neurons and muscle; and (5) calcium dyshomeostasis, driven by LRRK2/PINK1 mutations and STAT-PIAS2 pathway disruption, which further exacerbates mitochondrial swelling and neuronal excitotoxicity. These interconnected mechanisms converge on dopaminergic neuron degeneration in the substantia nigra, while muscle-specific OXPHOS deficits (e.g., enzyme complex I/III reduction) contribute to systemic PD pathology. The JAK-STAT-PIAS2 axis and IFNβ-IFNAR signaling are highlighted as modulators of neuronal survival and inflammatory responses.

Mitochondria play a critical role in the onset and progression of PD, with their dysfunction being closely associated with the pathological processes of PD. The following sections elaborate in detail on the specific manifestations and mechanistic roles of mitochondrial dysfunction in PD (Fig. 2). Firstly, as the “energy factories” of cells, mitochondria are primarily responsible for providing neurons with sufficient energy to maintain their normal physiological functions and various metabolic activities.^31^ In normal physiological states, mitochondria continuously synthesize ATP through a series of complex biochemical processes, such as oxidative phosphorylation (OXPHOS), providing the necessary energy support for neuronal electrical activity, neurotransmitter synthesis and release, etc.^32^ However, when mitochondria suffer from dysfunction, such as damage to their internal respiratory chain complexes, abnormal changes in membrane potential, or mutations in mtDNA, their ability to synthesize ATP significantly decreases, failing to meet the energy demands of neurons.^33^^,^^34^ Moreover, dysfunctional mitochondria release neurotoxic substances such as ROS radicals. These radicals, which have strong oxidizing properties, cause oxidative damage to various biomolecules within neurons, including lipids, proteins, and DNA, disrupting the normal structure and function of neurons.^35^ A key pathological feature of PD is the death of dopaminergic neurons in the substantia nigra. The aforementioned adverse consequences caused by mitochondrial dysfunction directly contribute to the gradual death of dopaminergic neurons, ultimately leading to the occurrence and progression of PD.

Moreover, from a genetic perspective, certain single-gene mutations are known to exist in PD, and these mutations are closely related to mitochondrial dysfunction and the degradation of damaged mitochondria. For example, the *PINK1* and *Parkin* genes encode proteins that normally participate in mitochondrial quality control mechanisms. When mitochondria are damaged and depolarized, the *PINK1* protein senses these changes and recruits the Parkin protein to the damaged mitochondria, initiating the marking of damaged mitochondria and subsequent autophagic degradation to eliminate those with abnormal functions and maintain the health of the mitochondrial population within the cell.^36^^,^^37^ However, in PD patients, mutations in these two genes prevent the normal recognition and degradation of damaged mitochondria, resulting in the accumulation of a large number of dysfunctional mitochondria within the cell. These mitochondria continuously release neurotoxic substances, exacerbating neuronal damage and ultimately triggering PD-related symptoms.^38^ However, the complete mechanisms underlying how these gene mutations precisely regulate mitochondrial dysfunction and subsequent degradation processes are still not fully understood in the scientific community and remain one of the key areas of in-depth exploration for many researchers.

## Mitochondrial dynamics impairment in PD

Mitochondrial dynamics, which encompass fission and fusion processes, are critical for maintaining mitochondrial integrity and neuronal survival (Fig. 2). In PD, dysregulation of these dynamics disrupts energy production, redox balance, and organelle quality control. Mitochondrial fission, mediated by dynamin-related protein 1 (Drp1) and fission 1 protein (FIS1), involves Drp1 oligomerization at the outer mitochondrial membrane (OMM) to fragment mitochondria. Excessive fission in PD models leads to mitochondrial fragmentation, reduced membrane potential, and impaired ATP synthesis, which are particularly detrimental to energy-dependent dopaminergic neurons.^39^, ^40^, ^41^ Conversely, mitochondrial fusion, which is regulated by OMM proteins Mfn1/2 and the inner membrane protein OPA1, ensures functional complementarity through the exchange of proteins, respiratory chain components, and mtDNA. Fusion defects in PD increase mtDNA mutation rates, disrupt OXPHOS, and increase ROS levels, exacerbating mitochondrial dysfunction and driving dopaminergic neuron degeneration.^42^^,^^43^

Mitochondrial dynamics are closely intertwined with mitophagy, a selective autophagic process that eliminates damaged mitochondria. The PINK1/Parkin pathway exemplifies this relationship: under physiological conditions, *PINK1* accumulates on depolarized mitochondria, recruiting *Parkin* to ubiquitinate OMM proteins (e.g., Mfn1/2), marking them for autophagic degradation via LC3-binding adaptors such as optineurin and nuclear dot protein 52 (NDP52).^38^^,^^44^^,^^45^ Genetic PD models highlight distinct dynamics-related pathologies. For instance, LRRK2-G2019S mutations induce hyperactive fission via Drp1 overactivation, impairing mitochondrial motility and mitophagy. Electron microscopy studies in transgenic mice and patient-derived disease-specific induced pluripotent stem cell (iPSC) dopaminergic neurons revealed fragmented mitochondria with reduced crista density, while Seahorse assays demonstrated impaired ATP production.^29^^,^^46^, ^47^, ^48^ Pharmacological inhibition of Drp1 (e.g., Mdivi-1) restored mitochondrial morphology and autophagic flux in these models.^49^^,^^50^ Conversely, *PINK1* deficiency causes fusion arrest, producing elongated mitochondria that are unable to compensate for damage.^51^ Mutant Parkin exacerbates dysfunction by ubiquitinating Mfn2, blocking fusion-dependent repair and increase ROS leakage.^52^ These dynamic defects intersect with calcium dysregulation: *PINK1* or *LRRK2* mutations elevate mitochondrial Ca^2+^ levels, disrupting ion homeostasis and inducing mitochondrial swelling, which accelerates neuronal death.^53^, ^54^, ^55^

Postmortem studies of PD patients revealed reduced *Parkin* activity in the substantia nigra and elevated mitochondrial proteins in autophagosomes, confirming mitophagy failure.^56^ In preclinical models, adeno-associated virus (AAV)-mediated *PINK1* delivery rescued mitophagy and reduced α-synuclein aggregation.^57^ Cerebrospinal fluid (CSF) analysis in early-stage PD patients revealed elevated mtDNA and decreased *PINK1*, suggesting that compromised mitophagy is a diagnostic marker.^58^ Innovative models, such as patient-derived iPSCs carrying LRRK2-G2019S, recapitulate PD pathology by generating dopaminergic neurons with senescence-associated degeneration, which is reversible via gene correction or LRRK2 kinase inhibitors.^59^, ^60^, ^61^, ^62^, ^63^ These findings align with postmortem brain studies showing nuclear membrane abnormalities,^62^ validating that mitochondrial dynamics is a therapeutic target. Future studies should prioritize translational validation, such as combining dynamic imaging (e.g., mito-QC reporters) with multi-omics profiling in patient cohorts, to refine therapeutic strategies. Mitochondrial dynamics impairment in PD involves a triad of fission/fusion imbalance, calcium overload, and mitophagy failure, synergistically driving dopaminergic neuron loss. Integrating genetic insights with iPSC models and targeted therapies offers a roadmap to restore mitochondrial homeostasis and halt disease progression.

## Mitochondrial dysfunction in sporadic PD (sPD)

sPD accounts for more than 90% of PD cases, and current research has found that its pathogenesis is closely related to the blockage of pathways regulating mitochondria, the powerhouses of neurons.^64^ When this pathway is blocked, a large number of damaged mitochondria accumulate continuously within the cell. Mitochondria, which serve as the “energy factories” of cells, normally produce sufficient energy for cells to maintain their various physiological activities. However, the accumulation of damaged mitochondria prevents them from effectively generating enough energy for cells, leading to neurons gradually losing their energy support and ultimately dying. Consequently, PD-related symptoms emerge.^64^^,^^65^

Notably, research on sporadic PD patients has also identified a crucial gene mutation, protein inhibitor of activated STAT (signal transducer and activator of transcription) 2 (PIAS2), which affects mitophagy, increasing the accumulation of aging mitochondria and oxidative stress. A series of experiments and analyses confirmed that the regulation of the Janus kinase (JAK)-STAT2-PIAS2 pathway plays a vital role in axonal growth, neuronal survival, and neuronal excitability.^66^ In patients with sPD, particularly those who progress to dementia (PDD), studies have found cytokine signaling dysregulation. The PIAS2 signaling pathway in PD involves multiple type I interferons (IFNs), their receptors, and downstream molecules, including dysregulation of the *IFNG*, *IFNGR1*, and *STAT4* genes, as well as up-regulation of negative regulators such as *PIAS2*, suggesting a blockade of the IFNβ-IFNAR signaling pathway.^66^^,^^67^ Sequence variations associated with *PIAS2* are linked to PD, and higher levels of PIAS2 mRNA and protein expression are observed in neurons of sPD patients. In mouse models, the overexpression of *PIAS2* leads to motor and cognitive impairments associated with the accumulation of phosphorylated α-synuclein and the loss of dopaminergic neurons.^68^ Studies have found that ectopic expression of *PIAS2* blocks mitophagy, resulting in the accumulation of senescent mitochondria and oxidative stress, disrupting the intracellular environment. *PIAS2* gene knockout restores mitochondrial homeostasis, oxidative stress, and the pERK1/2-pP53 signaling pathway, rescuing the clinicopathological manifestations of PDD in IFNβ^−/−^ mice.^66^^,^^69^ This indicates that *PIAS2* dysregulation promotes PD progression at multiple levels and *PIAS2* holds promise as a new therapeutic target for PD, especially for patients with PDD.

## Research progress on PD and mitochondrial dysfunction

Long-term studies of brain tissues in PD patients have consistently revealed prominent features of mitochondrial dysfunction. As early as 1990, a postmortem study comparing PD patients with age-matched controls demonstrated a significant reduction in the activity of mitochondrial complex I (NAD + CoQ reductase) in the substantia nigra of PD patients.^70^ Subsequent immunohistochemical analyses further confirmed that the staining intensity of this enzyme complex was specifically diminished in neuromelanin-containing neurons of the substantia nigra, while staining in the oculomotor nucleus and red nucleus within the same brain sections remained normal. Although age-related declines in nigral enzyme complex activity were observed in normal elderly individuals, the degree of reduction in PD patients was statistically distinct.^71^ Notably, these mitochondrial defects exhibited anatomical selectivity within the substantia nigra, predominantly localized to the pars compacta, with the reticulata remaining unaffected. The activities of other enzyme complexes (II, III, IV) and aconitase were preserved, and mitochondrial function in other brain regions (e.g., the cortex, cerebellum, and striatum) was not significantly different from that in controls.^72^^,^^73^

Recent advancements in multi-omics technologies have provided novel insights into mitochondrial abnormalities in PD. For instance, single-cell RNA sequencing of postmortem substantia nigra tissue from PD patients revealed cell-type-specific mitochondrial defects: dopaminergic neurons showed marked down-regulation of complex I subunits (NADH: ubiquinone oxidoreductase subunit B3 (NDUFB3), NDUFV1) and reduced expression of PINK1, while astrocytes exhibited impaired fatty acid oxidation-related genes.^74^, ^75^, ^76^ These findings align with earlier clinical observations of multi-complex OXPHOS defects in muscle biopsies from PD patients,^77^ collectively suggesting systemic mitochondrial dysfunction. Further experimental validation confirmed complex I deficiency in PD patient-derived neurons, which was partially rescued through yeast NADH dehydrogenase (NDI1) gene therapy.^78^ Additionally, the association between mtDNA damage signatures in microglia and neuroinflammation highlights the potential role of mitochondrial ROS in driving PD pathology.^79^

Muscle tissue studies also support systemic mitochondrial abnormalities in PD. For example, among six PD patients, five exhibited defects in multiple mitochondrial enzyme complexes in muscle tissue (only one patient was normal)^80^; however, no mtDNA deletions or point mutations were detected, and histochemical or electron microscopic examinations revealed no significant structural abnormalities. A detailed analysis of skeletal muscle mitochondrial function in eight PD patients demonstrated generalized reductions in enzyme complex activities (including complex V in two patients), while complexes II and III remained normal. Despite these defects, patients showed no overt muscle weakness, and mitochondrial oxygen consumption rates, blood biochemical markers (e.g., lactate and pyruvate), and histopathological findings displayed only mild nonspecific changes, such as type II fiber atrophy.^81^ These paradoxical observations suggest that mitochondrial dysfunction in PD may operate independently of traditional structural damage mechanisms.

In summary, mitochondrial dysfunction in PD manifests as both region-specific and systemic features, potentially serving as both a pathological driver and a biomarker. However, the underlying mechanisms, such as selective vulnerability, compensatory regulation, and their contributions to clinical symptoms, require further in-depth investigation.

## The relationship between aging and mitochondrial dysfunction in PD

Mitochondrial dysfunction plays a crucial role in the development of PD, and aging, as a significant risk factor for PD, is closely linked to mitochondrial dysfunction. As age increases, mitochondrial function gradually declines, which not only exacerbates mitochondrial dysfunction itself but also influences the progression of PD through a series of complex mechanisms. The following sections delve into the relationship between aging and mitochondrial dysfunction in PD.

## Comparison of the “genetic compensation” mechanism of mtDNA in brain cells between healthy elderly individuals and PD patients

Research has found that the brain cells of healthy elderly individuals possess a self-protection mechanism that compensates for damage caused by aging by generating more mtDNA.^82^ Mitochondria, as crucial “energy stations” of cells, play a vital role in maintaining normal mitochondrial function and ensuring the cellular energy supply, with their internal DNA serving as the foundation.^83^ During the natural process of aging, cells inevitably suffer damage from various internal and external factors. Healthy elderly individuals’ brain cells can partially repair and maintain mitochondrial function by increasing the production of mtDNA, thereby ensuring the continuation of normal cellular physiological activities.^84^^,^^85^ However, in the brain cells of PD patients, this protective mechanism that was originally used to compensate for aging-related damage is weakened. This leads to a decrease in the number of normal mtDNA, making mitochondria less equipped with sufficient “repair materials” and “coping strategies” when facing adverse effects caused by aging, such as free radical damage and metabolite accumulation, further disrupting their function.^86^^,^^87^ For instance, normally, sufficient mtDNA supports the normal expression of genes related to key structures and functions such as mitochondrial respiratory chain complexes, ensuring efficient respiratory chain operation for ATP synthesis.^88^ However, due to the reduction of normal mtDNA in PD patients, the function of respiratory chain complexes is impaired, resulting in decreased ATP production. Consequently, neurons cannot receive sufficient energy, making it difficult to maintain their normal physiological functions.^89^^,^^90^ This also becomes an important potential factor contributing to the development of PD.

## Aging triggers PD through multiple mechanisms

**Figure 3:**
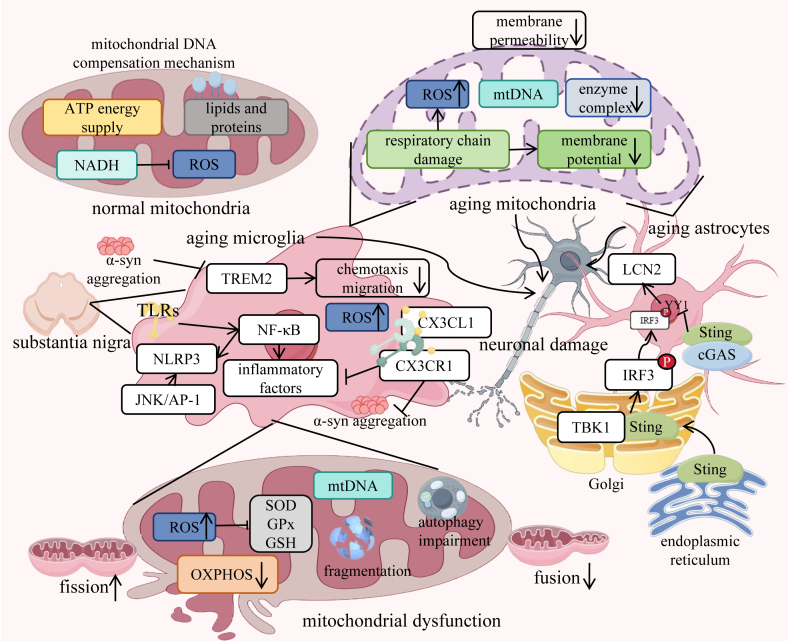
Aging exacerbates PD pathology through mitochondrial dysfunction and neuroinflammation. (1) Mitochondrial failure, where aging reduces compensatory mtDNA replication, leads to ROS accumulation, respiratory chain damage (NADH/CoQ deficiency), and membrane potential collapse. These defects impair ATP production while increasing oxidative damage to lipids/proteins. (2) α-Synuclein aggregation is amplified by aging microglia through TREM2 down-regulation and TLR/NF-κB/NLRP3 activation, which promotes the release of inflammatory factor (e.g., IL-1β and TNF-α) and JNK/AP-1 pathway-mediated neuronal stress. (3) Astrocyte senescence, driven by the cGAS–STING–IRF3 axis and LCN2 up-regulation, creates a neurotoxic environment via endoplasmic reticulum stress and diminished antioxidant defenses (SOD/GPx/GSH depletion). Collectively, these processes form a vicious cycle: mitochondrial OXPHOS fragmentation and calcium dyshomeostasis accelerate neuronal damage, while microglial/astrocytic inflammation further propagates α-synuclein pathology, ultimately driving dopaminergic neurodegeneration.

Aging drives the progressive loss of dopaminergic neurons in the substantia nigra through several synergistic pathways, with oxidative stress and mitochondrial dysfunction playing central roles (Fig. 3). As individuals age, the antioxidant defense system weakens, while ROS production increases, creating an oxidative imbalance. This oxidative stress damages critical cellular components in dopaminergic neurons, including mitochondrial membranes, mtDNA, and proteins involved in energy metabolism.^91^ The substantia nigra is particularly vulnerable due to its high basal oxidative load and dopamine metabolism, which generates reactive quinones. When mitochondria are compromised by oxidative attacks, their membrane potential collapses, respiratory chain activity declines, and ATP production falters.^92^ This energy crisis impairs neuronal function while generating even more ROS through electron leakage, establishing a self-perpetuating cycle of oxidative damage and bioenergetic failure that ultimately kills neurons.^48^^,^^93^, ^94^, ^95^

Mitochondrial deterioration is further compounded by age-related genomic instability. Accumulating mtDNA mutations disrupt OXPHOS, exacerbating ROS production. These ROS molecules then inflict additional mtDNA damage while simultaneously impairing the PINK1/Parkin mitophagy pathway, preventing the clearance of defective mitochondria.^96^ The situation worsens with decreasing NAD ^+^ levels during aging, which reduces sirtuin activity and compromises critical mitochondrial maintenance programs.^97^ Ca^2+^ dysregulation represents another key dimension of aging-related mitochondrial failure. Reduced expression of leucine zipper-EF-hand containing transmembrane protein 1 (LETM1) (a PINK1-regulated mitochondrial calcium transporter) leads to Ca^2+^ overload, triggering cristae swelling, excitotoxicity, and further impairment of ATP synthesis.^40^^,^^41^

In addition to these intrinsic mitochondrial defects, aging systematically undermines organelle function through multiple avenues: metabolic enzyme activity declines, membrane permeability increases, and protein quality control mechanisms falter.^98^, ^99^, ^100^ In dopaminergic neurons, these changes are particularly catastrophic. The high energy demands of maintaining spontaneous pacemaking activity, synthesizing and releasing dopamine, and supporting extensive axonal arbors make these neurons highly sensitive to mitochondrial insufficiency.^101^ When aging mitochondria can no longer meet these demands, electrical activity becomes erratic, dopamine homeostasis is disrupted, and neurons gradually succumb to metabolic collapse.^102^^,^^103^ Critically, this mitochondrial dysfunction is both driven and exacerbated by the aging process itself—a vicious cycle that accelerates PD pathogenesis.

This multifaceted interplay between aging and mitochondrial failure creates a biological “perfect storm” in the substantia nigra, where cumulative damage across molecular, organellar and cellular scales conspires to produce the characteristic neurodegeneration of PD. Understanding these mechanisms not only clarifies disease etiology but also highlights promising therapeutic targets, from increasing mitophagy to modulating calcium homeostasis or enhancing antioxidant defenses.

## Aging microglia and PD

Microglia, key immune cells in the CNS, maintain neural homeostasis by regulating neuronal survival, clearing apoptotic cells, and supporting neurogenesis while also mediating immune surveillance via inflammatory mediator release.^104^, ^105^, ^106^ In PD, their role shifts dramatically: aberrantly activated microglia drive neuroinflammation, releasing factors that induce neuronal injury, apoptosis, and degeneration, exacerbating disease progression.^107^ Understanding how aging-related molecular defects, particularly mitochondrial dysfunction, in microglia contribute to PD pathogenesis is critical for developing targeted therapies, a rapidly evolving research frontier.

## Morphological and functional alterations of aging microglia

The morphological changes of aging microglia include debranching and fragmentation of protrusions. Normally, the branched structure of microglia aids in monitoring changes within the CNS. However, cellular aging leads to a reduction in branches, narrowing the scope of immune monitoring.^108^ For instance, in the substantia nigra of patients with PD, microglia exhibit decreased branching, making neurons more vulnerable. The ability of microglia to clear neurotoxic substances correlates with their morphology. Fragmentation of protrusions due to aging reduces clearance efficiency. *In vitro* experiments have shown that aging microglia exhibit a decreased ability to clear α-synuclein aggregates, leading to the accumulation of neurotoxic substances and deterioration of the neuronal environment.^109^

Spatial transcriptomics has unveiled the spatial heterogeneity and central role of aging microglia in PD pathogenesis. In the substantia nigra of elderly PD patients, microglia exhibit marked pathological remodeling: morphological collapse characterized by dendritic retraction and somatic hypertrophy in α-synuclein-enriched regions correlates with local neurodegeneration.^110^^,^^111^ This pathological state coincides with metabolic reprogramming, featuring a characteristic shift from mitochondrial OXPHOS to glycolysis, where up-regulated hexokinase 2 (HK2) and pyruvate dehydrogenase kinase 2 (PDK2) not only drive energy metabolism dysregulation but also amplify neuroinflammatory responses.^112^ Mechanistic studies revealed that down-regulation of triggering receptor expressed on myeloid cells 2 (TREM2) in nigral microglia triggers dual pathogenic cascades: impaired phagocytic clearance of α-synuclein aggregates^113^ and activation of the nuclear factor kappa B (NF-κB)/NOD-like receptor thermal protein domain associated protein 3 (NLRP3) inflammasome axis, promoting aberrant interleukin-1β (IL-1β) secretion.^114^ Notably, TREM2 overexpression in SNCA-A53T transgenic mouse models restored microglial synaptic pruning capacity and reduced dopaminergic neuron loss, identifying TREM2 as a critical therapeutic target for modulating neuroinflammation in PD.^115^

TREM2 dysfunction exhibits multidimensional connections with PD progression. In aged mouse models, the significantly reduced TREM2 expression in substantia nigra microglia drives a phenotypic shift from anti-inflammatory to pro-inflammatory activation, exacerbating neuroinflammation in the nigrostriatal pathway and α-synuclein deposition.^115^^,^^116^ This functional impairment further disrupts cellular behavior through molecular networks: TREM2 deficiency down-regulates chemotaxis-related genes (*INPP5D*, *CSF3R*, *P2RY6*, etc.), impairing microglial directional migration and clearance of apoptotic neurons, thereby causing abnormal accumulation of neurotoxic substances in the substantia nigra.^117^ Age-dependent TREM2 attenuation is particularly pronounced in PD animal models, where aged mice display markedly reduced phagocytic efficiency toward α-synuclein aggregates compared to their younger counterparts. This dysfunction directly accelerates pathological α-synuclein accumulation, triggering the degenerative death of dopaminergic neurons.^118^ In summary, aging microglia establish a vicious cycle between α-synuclein pathology and neuroinflammation through spatially specific morphological, metabolic, and functional remodeling. As a central regulatory hub, TREM2 expression levels not only determine microglial phagocytic efficiency but also influence PD neurodegenerative progression by modulating chemotaxis networks and inflammatory phenotypes. These findings provide a theoretical foundation for developing precision therapies targeting the neuroimmune microenvironment.

The C-X3-C motif chemokine ligand 1 (CX3CL1)/C-X3-C chemokine receptor 1 (CX3CR1) signaling pathway is a crucial pathway for maintaining immune homeostasis. CX3CL1 can mitigate the toxic effects of α-synuclein on dopaminergic neurons and is closely related to the regulation of neuroprotection and synaptic plasticity.^119^^,^^120^ As cellular aging progresses, the number of CX3CR1 receptors on the cell surface decreases along with the down-regulation of CX3CL1 ligand expression levels in the body. The overall down-regulation of this signaling pathway leads to an increase in the pro-inflammatory cytokine IL-1β secreted by microglia.^121^ This may be one of the driving factors for neuroinflammation induced by aging microglia. Pathological α-synuclein can induce microglia to differentiate into a pro-inflammatory phenotype through the Toll-like receptor (TLR) 1/2-mediated NF-κB signaling pathway, releasing neurotoxic substances such as nitric oxide (NO) and ROS, as well as pro-inflammatory cytokines such as tumor necrosis factor-α (TNF-α), IL-1, and IL-6. This exacerbates oxidative stress, thereby inducing neuroinflammation, promoting microglial senescence, affecting their normal functions, and driving the progression of PD.^122^^,^^123^

## Mitochondrial dysfunction in aging microglia

Mitochondrial dysfunction in aging microglia primarily manifests as two aspects: mitochondrial energy metabolism disorder and abnormal mitochondrial quality control. Under normal physiological conditions, mitochondria synthesize ATP through the tricarboxylic acid cycle and OXPHOS to provide energy for various cellular activities.^124^ However, in aging microglia, the activity of mitochondrial respiratory chain complexes significantly decreases, such as a reduction in complex I activity, leading to impedance in the electron transfer process, decreased ATP synthesis, and an insufficient cellular energy supply.^125^ This energy metabolism disorder affects the normal function of microglia, impairing their ability to respond timely and effectively to external stimuli.

Mitochondrial quality control is a crucial mechanism for maintaining normal mitochondrial function, encompassing processes such as mitochondrial fission, fusion, and autophagy.^126^, ^127^, ^128^ In aging microglia, the balance between mitochondrial fission and fusion is disrupted, often resulting in abnormal mitochondrial morphology and fragmentation. Additionally, impaired mitochondrial autophagy fails to promptly clear damaged mitochondria, leading to their accumulation within the cell and further exacerbating mitochondrial dysfunction. These dysfunctions not only impact the normal metabolism and function of microglia themselves but also adversely affect surrounding neurons, promoting the pathological progression of PD. Studies have shown that the inflammatory factors released by aging microglia increase due to mitochondrial dysfunction, thereby intensifying oxidative stress damage to neurons and causing degeneration and death of dopaminergic neurons.^129^

In the process of aging microglia participating in the pathogenesis of PD, multiple signaling pathways play pivotal roles, with the activation of the c-Jun N-terminal kinase (JNK)/activator protein 1 (AP-1) and NF-κB signaling pathways being particularly significant. The JNK/AP-1 signaling pathway has important functions in physiological and pathological processes such as the cell cycle, growth, apoptosis, and stress.^130^ In PD, the activation of the JNK/AP-1 signaling pathway can lead to a series of reactions, including the reduction of mitochondrial complex I, the release of cytochrome C, and increased intracellular reactive oxygen species, ultimately causing dysfunction and even apoptosis of dopaminergic neurons.^131^ Furthermore, it drives the activation of NLRP3 inflammasome, mediating neuroinflammatory responses, which are closely related to the occurrence and development of PD.^132^ Studies on PD model mice have found that the JNK inhibitor SP600125 can significantly reduce the expression levels of key proteins in the JNK/AP-1 signaling pathway, key proteins in the NLRP3 inflammasome, and inflammatory factors in microglia, indicating that the JNK/AP-1 signaling pathway plays a crucial driving role in the neuroinflammatory responses mediated by aging microglia.^133^ A deeper understanding of these mechanisms of aging microglia in PD is of great significance for exploring new diagnostic and therapeutic targets, and thus better addressing PD as an aging-related neurodegenerative disease.

## Aging astrocytes and PD

### Morphological and functional changes in aging astrocytes

Aging astrocytes undergo morphological and functional decline, with reduced branching, enlarged/flattened cell bodies, and diminished metabolic capacity, impairing the glucose supply to neurons and weakening antioxidant defenses, thereby increasing oxidative stress and biomacromolecule damage.^134^^,^^135^ In PD, these changes disrupt neurotransmitter homeostasis (e.g., elevated synaptic glutamate, a neurotoxin), reduce neurotrophic factor secretion critical for synaptic maintenance, and promote inflammatory factor release, collectively creating a hostile microenvironment that exacerbates dopaminergic neuron vulnerability and degeneration.^136^^,^^137^

### The cGAS–STING–YY1 axis signaling pathway

The cGAS–STING–-YY1 axis emerges as a critical mechanistic link between mitochondrial dysfunction, neuroinflammation, and PD progression (Fig. 3). In aging or damaged cells, mtDNA leaks into the cytoplasm due to impaired membrane integrity, where it is recognized by cyclic GMP AMP synthase (cGAS). This triggers cGAS activation and the synthesis of cyclic GMP AMP (cGAMP), which binds and activates STING. Activated STING translocates to the Golgi apparatus, where it recruits TANK-binding kinase 1 (TBK1) to phosphorylate interferon regulatory factor 3 (IRF3). Phosphorylated IRF3 dimerizes and translocates to the nucleus, where it drives the expression of pro-inflammatory genes (e.g., IFN-β) that exacerbate neuroinflammation.^138^, ^139^, ^140^

Concurrently, STING activation inhibits the nuclear translocation of the transcription factor YY1. Under physiological conditions, YY1 suppresses expression of the pro-senescence factor lipocalin-2 (LCN2). When sequestered in the cytoplasm by STING, the YY1-mediated repression of LCN2 is lifted, leading to LCN2 up-regulation. Elevated LCN2 promotes astrocyte senescence, characterized by reduced metabolic support, increased secretion of neurotoxic cytokines (e.g., IL-6, TNF-α), and impaired synaptic maintenance.^141^ These senescent astrocytes create a hostile microenvironment that accelerates dopaminergic neuron degeneration, a hallmark of PD pathology.

Notably, preclinical studies have demonstrated that pharmacological inhibition of the cGAS–STING pathway reduces astrocyte senescence, mitigates neuroinflammation, and preserves motor function in PD models. For instance, genetic ablation of STING in aged mice attenuates LCN2-driven astrocyte senescence and protects nigrostriatal neurons.^141^ These findings position the cGAS-STING-YY1 axis as a promising therapeutic target, with potential interventions including small-molecule STING inhibitors or strategies to increase YY1 nuclear localization.

### Mitochondrial dysfunction in aging astrocytes

Mitochondria produce a small amount of ROS during electron transport in the respiratory chain, and the intracellular antioxidant system is responsible for scavenging ROS to maintain redox balance. However, when aging astrocytes lead to mitochondrial dysfunction, ROS production increases, antioxidant system imbalance occurs, and oxidative stress levels rise. The activity of respiratory chain complexes decreases, leading to increased electron leakage and the generation of more ROS. The activities of antioxidant enzymes such as SOD and glutathione peroxidase (GPx) decrease, and the levels of antioxidant substances such as glutathione (GSH) decrease, making it impossible to effectively scavenge ROS.^142^^,^^143^ Oxidative stress can cause mitochondrial membrane lipid peroxidation, protein oxidative damage, and mtDNA mutations, further impairing mitochondrial function and creating a vicious cycle. In the brains of patients with PD, ROS produced by aging astrocytes can diffuse to neurons, damaging their cell membranes, proteins, and DNA and triggering oxidative stress damage and death.^144^ In the brains of PD patients, the fragmentation of mitochondria in aging astrocytes significantly increases, closely related to neuronal degeneration and death.^145^^,^^146^ Additionally, abnormal protein synthesis may affect other mitochondrial functions, such as maintenance mitochondrial morphology and material transport, further exacerbating mitochondrial dysfunction and having a negative impact on the function of aging astrocytes in PD (Fig. 3). In summary, future research on aging astrocytes and mitochondrial dysfunction in PD patients will develop in a multidimensional and refined direction. Through the close integration of basic research and clinical applications, it is hoped that more effective treatment methods could be developed, bringing new hope to PD patients.

### Conclusions

PD is a complex neurodegenerative disorder characterized by progressive motor and non-motor symptoms that significantly impact patients’ quality of life. While its exact pathogenesis remains incompletely understood, several key pathological mechanisms have been identified. Mitochondrial dysfunction emerges as a central player, contributing to neuronal energy failure and death through impaired autophagy (e.g., PINK1/Parkin pathway defects), oxidative stress, and disrupted calcium homeostasis. These mitochondrial abnormalities are further exacerbated by genetic risk factors (e.g., *SNCA* and *LRRK2* mutations) and aging-related processes.

Importantly, recent studies highlight the crucial role of aging glial cells in PD progression. Microglia undergo senescence-associated changes, including TREM2 down-regulation and impaired phagocytosis, leading to neurotoxic protein accumulation. Similarly, aging astrocytes exhibit mitochondrial dysfunction and activate the cGAS–STING–YY1 pathway, promoting neuroinflammation. These findings suggest that therapeutic strategies should simultaneously target both neuronal and glial pathologies. Promising approaches include enhancing mitochondrial quality control (e.g., PINK1/Parkin activators), modulating neuroinflammation (e.g., TREM2 agonists and cGAS-STING inhibitors), and developing PIAS2-targeted therapies for specific PD subtypes.

In the future, several research directions warrant priority. First, advanced single-cell technologies should be employed to map mitochondrial–glial–neuronal interactions across disease stages. Second, the development of clinically relevant biomarkers (e.g., mtDNA damage signatures) could enable earlier diagnosis. Finally, interdisciplinary efforts combining gene editing, nanotechnology, and precision medicine approaches may yield breakthrough therapies. While challenges remain, these strategies offer realistic pathways to develop effective treatments that address both the neuronal and glial aspects of PD pathology.

## CRediT authorship contribution statement

**Tingting Liu:** Writing – original draft. **Jingwen Li:** Writing – original draft. **Haojie Wu:** Writing – original draft. **Junbo Qiao:** Funding acquisition, Supervision, Writing – review & editing. **Jianshe Wei:** Writing – review & editing.

## Funding

This work was supported partly by the 10.13039/501100001809National Natural Science Foundation of China (No. 32161143021, 81271410), Henan University graduate “Talent Program” of Henan Province, China (SYLYC2023092), 10.13039/501100006407Henan Natural Science Foundation of China (No. 182300410313), and Key Research and Development Project of Henan Province, China (No. 231111311400).

## Conflict of interests

The authors declared that they have no competing interests.
