# Supplementary figures and images for: Unraveling Parkinson's disease: The mystery of mitochondria and the role of aging
Source: Genes Dis. 2025 Jun 10;13(2):101719. doi: 10.1016/j.gendis.2025.101719 (PMC12719679; doi:10.1016/j.gendis.2025.101719)

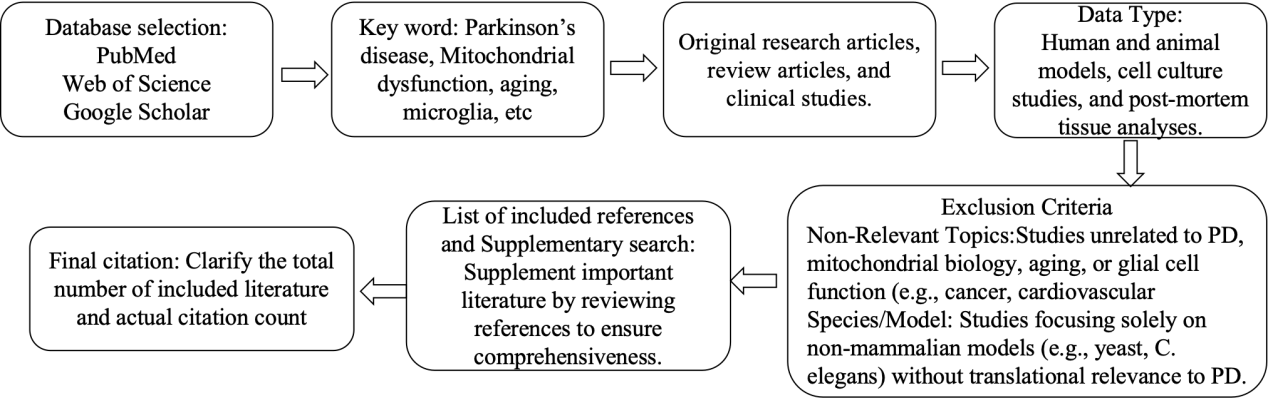


**Figure S1** Flowchart: Literature Search and Selection Process

Supplement: Multimedia component 1 [file mmc1.docx]
